# RSK2-Mediated ELK3 Activation Enhances Cell Transformation and Breast Cancer Cell Growth by Regulation of c-fos Promoter Activity

**DOI:** 10.3390/ijms20081994

**Published:** 2019-04-23

**Authors:** Sun-Mi Yoo, Cheol-Jung Lee, Hyun-Jung An, Joo Young Lee, Hye Suk Lee, Han Chang Kang, Sung-Jun Cho, Seung-Min Kim, Juhee Park, Dae Joon Kim, Yong-Yeon Cho

**Affiliations:** 1 Integrated Research Institute of Pharmaceutical Sciences, BRL & BK21 Plus team, Pharmaceutical Biochemistry, College of Pharmacy, The Catholic University of Korea, 43, Jibong-ro, Wonmi-gu, Bucheon-si, Gyeonggi-do 14662, Korea; apple_ma@naver.com (S.-M.Y.); veritas0613@naver.com (C.-J.L.); anhyunjung11@gmail.com (H.-J.A.); joolee@catholic.ac.kr (J.Y.L.); sianalee@catholic.ac.kr (H.S.L.); hckang@catholic.ac.kr (H.C.K.); sonatamao@naver.com (S.-M.K.); pjhlove6385@naver.com (J.P.); 2 Department of Integrative Biology and Physiology, University of Minnesota, Minneapolis, MN 55455, USA; choxx500@umn.edu; 3 Department of Molecular Science, School of Medicine, University of Texas Rio Grande Valley, Edinburg Research Education Building, Room 2.112, 1214, W. Schunior St, Edinburg, TX 78541, USA; dae.kim@utrgv.edu

**Keywords:** RSK2, ELK3, protein–protein interaction, cell signaling, cell transformation

## Abstract

Ribosomal S6 kinase 2 (RSK2), regulated by Ras/Raf/MEKs/ERKs, transmits upstream activation signals to downstream substrates including kinases and transcription and epigenetic factors. We observed that ELK members, including ELK1, 3, and 4, highly interacted with RSK2. We further observed that the RSK2-ELK3 interaction was mediated by N-terminal kinase and linker domains of RSK2, and the D and C domains of ELK3, resulting in the phosphorylation of ELK3. Importantly, RSK2-mediated ELK3 enhanced *c-fos* promoter activity. Notably, chemical inhibition of RSK2 signaling using kaempferol (a RSK2 inhibitor) or U0126 (a selective MEK inhibitor) suppressed EGF-induced *c-fos* promoter activity. Moreover, functional deletion of RSK2 by knockdown or knockout showed that RSK2 deficiency suppressed EGF-induced *c-fos* promoter activity, resulting in inhibition of AP-1 transactivation activity and Ras-mediated foci formation in NIH3T3 cells. Immunocytofluorescence assay demonstrated that RSK2 deficiency reduced ELK3 localization in the nucleus. In MDA-MB-231 breast cancer cells, knockdown of RSK2 or ELK3 suppressed cell proliferation with accumulation at the G1 cell cycle phase, resulting in inhibition of foci formation and anchorage-independent cancer colony growth in soft agar. Taken together, these results indicate that a novel RSK2/ELK3 signaling axis, by enhancing c-Fos-mediated AP-1 transactivation activity, has an essential role in cancer cell proliferation and colony growth.

## 1. Introduction

The E26 transformation-specific (ETS) proteins form a large family of transcription factors that regulate various functions in cells [1] through the formation of ternary complex factors (TCFs) with serum response factor and serum response elements [2,3]. ELK3, also known as Net, SAP-2, or Erp, is a member of the ETS transcription factor family and is distinguished from other members of the ETS family by its ability to repress transcription [4]. Although ELK3 was initially identified as a transcriptional repressor of *c-fos* gene expression [5], it also has a role as a transactivator when it has been phosphorylated by the Ras-mediated mitogen-activated protein kinase (MAPK) signaling pathway [6]. Generally, the MAPK signaling pathway is upregulated by diverse stimuli including growth factors, such as EGF, environmental stresses, such as ultraviolet light, as well as cytokines and other factors, depending on the cellular context [7,8]. Activation signals initiated from the cytoplasmic membrane transduce to the nucleus through the phosphorylation conveyer cascade system [9]. In the nucleus, transcription factors are eventually activated, resulting in the regulation of various cellular behaviors including cell proliferation, transformation, migration, and death [10].

The MAPK signaling pathway is composed of extracellular signal-regulated kinases (ERK), c-Jun N-terminal kinases (JNK) and p38 MAP kinases (p38) [11]. Traditionally, the ERK signaling pathway has an essential role in cell proliferation and cell transformation, whereas JNK and p38 kinase signaling are reported to modulate the inflammatory response and environmental stress [12,13]. Our research group has focused mainly on the signaling axis mediated by ERK, which is known as an upstream kinase of ELK [14,15,16]. However, accumulating data have indicated that ELK1 is activated by MAPK including ERK, JNK, and p38, whereas ELK3 and ELK4 are activated by ERK and p38 [15,17,18,19,20]. Moreover, ERKs spontaneously bind with RSKs when cells enter a quiescence stage [21,22,23]. When cells are stimulated with growth factors, phosphorylated ERK1/2 through the Ras/MEK signaling pathway transduces activation signals to p90 ribosomal S6 kinases (RSKs) via phosphorylation [22,24]. Moreover, recent in vitro kinase assay results demonstrated that ERK1/2, but not p38 kinases, phosphorylates RSK2 and acts as an upstream kinase of RSK2 [25]. Based on the activation of RSKs, the N-terminal kinase domain of RSKs induces autophosphorylation at the ERK docking site located in the C-terminal domain of RSKs [26], resulting in the dissociation of ERK1/2 from RSK [23]. Importantly, although RSK1 and RSK2 have no nuclear localization signals in their polypeptides, activated RSK2 has been detected in the nucleus [27]. Unfortunately, molecular mechanisms for the nuclear localization of RSK1 and RSK2 have not been fully elucidated.

ELK3 is activated by MAPK-associated pathways [6], and it has an important role in various physiological processes, including cell migration, invasion, wound healing, angiogenesis, and tumorigenesis, by regulating c-Fos, early growth response protein 1 (egr-1) [28], and plasminogen activator inhibitor-1 (PAI-1) [29]. Moreover, in mouse hepatocytes, ELK3-mediated egr-1 regulation has an important role in the epithelial-mesenchymal transition (EMT) [30,31], a critical event in the process of cancer invasion and metastasis. Recently, it was demonstrated that ELK3 regulates hypoxia-induced factor 1α (HIF-1α); HIF-1α is a transcription factor that has an essential role in the regulation of genes associated with cancer metastasis, invasion, angiogenesis, cellular proliferation, apoptosis, and glucose metabolism [32,33]. Moreover, HIF-1α-mediated vascular endothelial growth factor and metalloproteinase-2 have been linked to the development, invasion, and metastasis of hepatocellular carcinoma [34,35]. Importantly, in vivo studies of the function of ELK3 in carcinogenesis have demonstrated that ELK3 deficient mice have smaller tumors as a result of impairment of vascularization and oxygenation [36]. Our research group previously demonstrated that RSK2 deficiency impairs cell migration and invasion through the inhibition of MMP-2 and MMP-9 gene expressions [37]. However, a direct relationship between RSK2 and ELK3 has not yet been elucidated.

## 2. Results

### 2.1. ELKs Are Novel Binding Partners with RSK2

**Figure 1:**
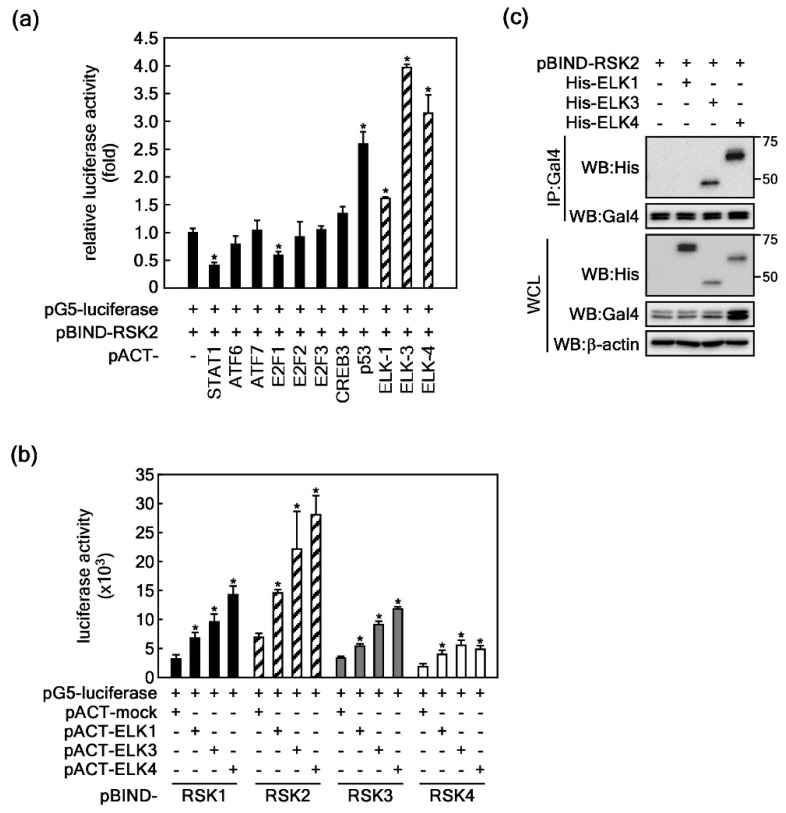
Ribosomal S6 kinase 2 (RSK2) is a novel ELK binding partner. (**a**) Mammalian two-hybrid assay. At the same molar ratio, each of 29 transcription factors cloned in pACT mammalian two-hybrid vectors was introduced into HEK293T cells with pBIND-RSK2 and pG5-luciferase reporter plasmid in wells of 48-well plates. The total amount of the three vectors did not exceed 100 ng. Firefly luciferase activity was normalized to *Renilla* luciferase activity. Data were obtained from three independent experiments, and values are presented are means ± SEM. * *p* < 0.01 versus pACT-mock control cells (Student’s *t*-test). (**b**) Comparison of binding affinity of ELKs with RSKs. At the same molar ratio, each ELK1, 3, and 4 in pACT, and each RSK1, 2, 3, and 4 in the pBIND mammalian two-hybrid vectors was introduced into HEK293T cells with pG5-luciferase reporter plasmid into wells of 48-well plates. Firefly luciferase activity was normalized to *Renilla* luciferase activity. Data were obtained from three independent experiments, and the values presented are means ± SEM. * *p* < 0.05 versus pACT-mock control cells (Student’s *t*-test). WCL, whole cell lysate; IP, immunoprecipitation. (**c**) Representative images used to confirm that RSK2 binds with ELKs. The pBIND-RSK2 and each of pcDNA4-HisMax-ELK1, -ELK3 and -ELK4 were co-expressed in HEK293T cells. The binding of RSK2 and each of the ELKs was visualized with IP and western blotting using specific antibodies as indicated.

The results in our previous study demonstrated that RSKs, including ribosomal S6 kinase 2 (RSK2), are located downstream of ERKs in the MAPK signaling pathway, and that ERK and RSK are spontaneously bound in the cytoplasm [25]. Moreover, our research group has suggested that RSK2 might act as a hub to transduce Ras-mediated ERK signaling [38] to downstream target proteins such as p53, c-Fos, NFAT3, ATF-1, and histone H3, resulting in modulation of diverse cellular processes including cell proliferation [38], transformation [38], cell migration and invasion [37], cell survival [39], and protein synthesis [40]. However, although ELK3 is classically regulated by the Ras-ERK signaling pathway [38], the direct upstream kinases of ELKs, especially those of ELK3, are unclear. To identify the binding partners of transcription factors for RSK2, we conducted mammalian two-hybrid assays using 29 pACT-transcription factors and pBIND-RSK2 as bait in HEK293T cells. We found that ELK family members including ELK1, 3, and 4 exhibited higher protein-protein interactions with RSK2 than with other transcription factors (Figure 1a). The interactions of ELK3 and ELK4 with RSK2 were higher than those of the already known RSK2 substrates CREBs and p53, results for which were published by our research group [41,42] (Figure 1a). Based on the high interaction levels, we focused on ETS transcription factors. To compare the binding strengths between each RSK member and ELK1, 3, or 4, we conducted further mammalian two-hybrid assays. The results showed that, compared to RSK1, 3, and 4, RSK2 more strongly interacted with ELKs, whereas RSK1 had a moderate interaction with ELKs, and RSK3 and RSK4 had weak interactions with ELKs (Figure 1b). Since our research group had conducted previous research into the RSK2 signaling network and cell proliferation and transformation, we further investigated the actual binding by examining immunoprecipitation results using cells that transiently expressed RSK2 and each of ELK1, 3, and 4 in HEK293T cells. We observed that ELK3 and ELK4 were strongly bound to RSK2, whereas ELK1 were relatively weakly bound to RSK2 (Figure 1c). These results indicated that RSK2 is a novel binding partner of ELKs.

### 2.2. ELK3 Is a Substrate of RSK2

**Figure 2:**
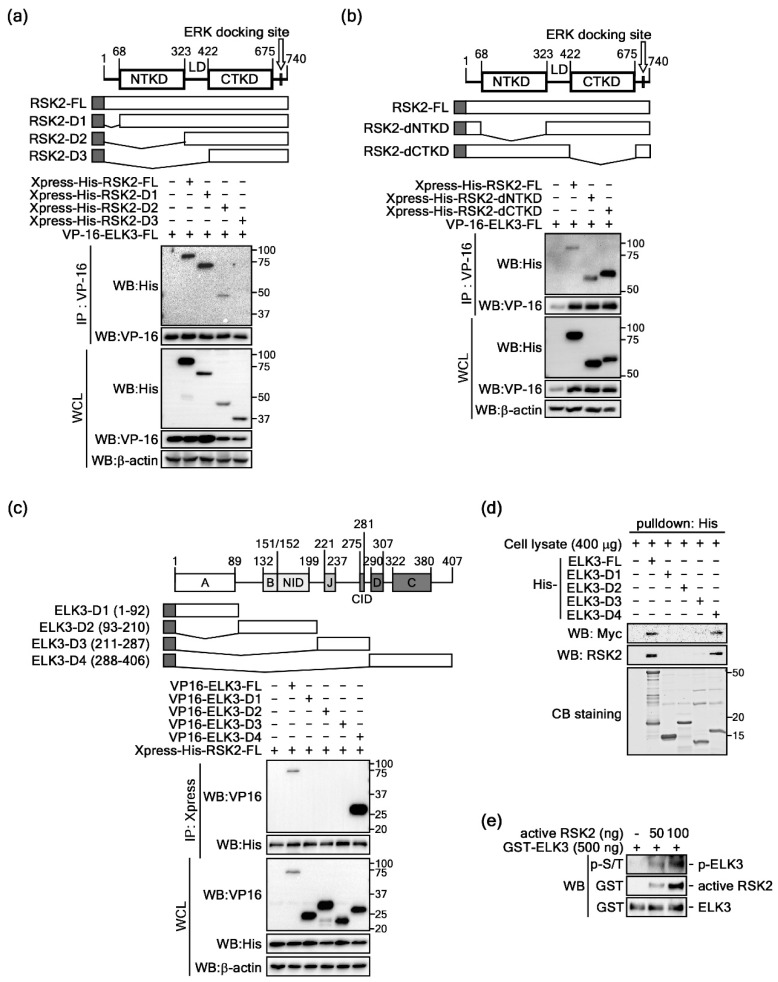
Determination of the binding domain for the RSK2-ELK3 interaction. (**a**) Representative images of immunoprecipitation results for RSK2 serial deletion mutants with full-length ELK3. The expression constructs were co-expressed in HEK293T cells as indicated, and ELK3 was immunoprecipitated with a VP-16 tag specific antibody. Binding of the RSK2 serial deletion mutants was visualized by western blotting using His-tag specific antibodies. WCL, whole cell lysate; IP, immunoprecipitation. (**b**) Representative IP images of RSK2 kinase deletion mutants with full-length ELK3. RSK2 kinase deletion mutants were co-expressed in HEK293T cells as indicated and ELK3 was immunoprecipitated with VP-16 tag specific antibody. The binding of RSK2 kinase deletion mutants was visualized by western blotting using His-tag specific antibody. WCL, whole cell lysate; IP, immunoprecipitation. (**c**) Representative IP images of ELK3 serial deletion mutants with full-length RSK2. The expression constructs were co-expressed in HEK293T cells as indicated, and RSK2 was immunoprecipitated with an Xpress tag specific antibody. The binding of ELK3 serial deletion mutants was visualized by western blotting using VP-16 tag specific antibody. WCL, whole cell lysate; IP, immunoprecipitation. (**d**) Representative images of pulldown assay results. Bacterially expressed His-ELK1-FL, -D1, -D2, -D3, and –D4 were coupled with Ni-IDA and then combined with cell lysates of HEK293T cells transiently expressing Myc-RSK2. The RSK2 binding was determined by pulldown of the Ni-IDA bead by using western blotting with Myc and RSK2 antibodies as indicated. CB staining, Coomassie blue staining. (**e**) Representative images of the in vitro kinase assay results. Commercially active RSK2 was combined with GST-ELK3 purified from *Escherichia coli* with cold ATP. The in vitro kinase assay was conducted at 30 °C for 30 min. The phosphorylated ELK3 was visualized by western blotting using a phospho-S/T specific antibody.

Previous results indicated that ELKs are binding partners for RSK2 (Figure 1). Although the domain structures and functions of the ETS family have been well-characterized, ELK1, 3, and 4 are relatively less well elucidated. Amino acid alignments of ELK1 (GenBank accession #: CAG47048), ELK3 (GenBank accession # CAG47047) and ELK4 (GenBank accession # EAW91574) indicate that ELKs harbor about 25.5% amino acid homology. However, we observed that A (ETS domain), B (SRF interaction domain) and C (is responsible for transcriptional activity and contains MAP kinase phosphorylation site) domains hade higher levels of amino acid homology among ELK1, 3, and 4 (Supplementary Figure S1). However, other domains including NID (N-terminal inhibitory domain), J domain (JNK docking site), CID (repression domain) and D (MAP kinase docking site) domains had very low amino acid homology (Supplementary Figure S1). Moreover, we analyzed the EST gene expression profiles of human RSKs and ELKs in different organ tissues as provided by UniGene (https://www.ncbi.nlm.nih.gov/unigene/?term, gene symbols for RSK1, RPS6KA1; RSK2, RPS6KA3; RSK3, RPS6KA2; RSK4, RPS6KA6; ELK1, ELK1; ELK3, ELK3; ELK4, ELK4). The results showed that RSK1-4 and ELK1-3 were widely and differentially expressed in different organ tissues (Supplementary Table S1). The organ tissues harboring the highest levels of RSK2 transcripts among RSKs were bone, liver, muscle, pharynx, skin, and vascular (Supplementary Table S1). Moreover, ELK3 was highly detected in adrenal gland, bone marrow, ear, esophagus, heart, liver, lymph node, mouth, pharynx, skin, trachea, umbilical cord, uterus, and vascular tissues (Supplementary Table S1). To analyze the binding domain of RSK2 and ELK3, we conducted immunoprecipitation using cells co-expressing RSK2 serial deletion mutants and full-length ELK3 (Figure 2a). The results indicated that the strong binding of RSK2-FL and RSK2-D1 with ELK3 was reduced in RSK2-D2 (Figure 2a), indicating that the N-terminal kinase domain (NTKD) of RSK2 might be involved in the interaction of RSK2 with ELK3. Moreover, RSK2 and ELK3 binding were absent in RSK2-D3, in which the linker domain (LD) was deleted, indicating that the LD of RSK2 has an important role in the interaction of RSK2 and ELK3 (Figure 2a). Since our previous studies have indicated that NTKD has an essential role in substrate phosphorylation, we needed to confirm the role of RSK2 NTKD in the RSK2 and ELK3 interaction. Thus, we constructed kinase domain-deleted RSK2 constructs (Figure 2b). Immunoprecipitation results demonstrated that the RSK2-dNTKD was weakly bound to ELK3 compared to the binding level of RSK2-dCTKD (Figure 2b), indicating that the NTKD of RSK2 has a key role in RSK2 and ELK3 interaction. On the other hand, we further analyzed the ELK3 binding domain to RSK2 using ELK3 deletion mutants (Figure 2c). We observed that only ELK3-D4 harboring aa 288-406, D and C domains, and variable domain III was co-immunoprecipitated (Figure 2c). Moreover, Ni-IDA pulldown assays using His-ELK3-FL, -D1, -D2, -D3 and –D4 expressed in bacteria and Myc-RSK2 expressing HEK293T cell lysate demonstrated that ELK3-D4 interacted with RSK2 (Figure 2d). An in vitro kinase assay using an active RSK2 and *Escherichia coli* purified GST-ELK3 showed phosphorylation of ELK3 when using the antibody recognizing phospho-Ser/Thr and indicated a dose-dependent increase of active RSK2 protein-enhanced phosphorylation of ELK3 (Figure 2e). These results demonstrated that RSK2 phosphorylates ELK3 by interaction through the LD and NTKD of RSK2 and the D and C domains of ELK3.

### 2.3. RSK2-Mediated ELK3 Transactivation Activity Regulates Ras-Mediated Cell Transformation

**Figure 3:**
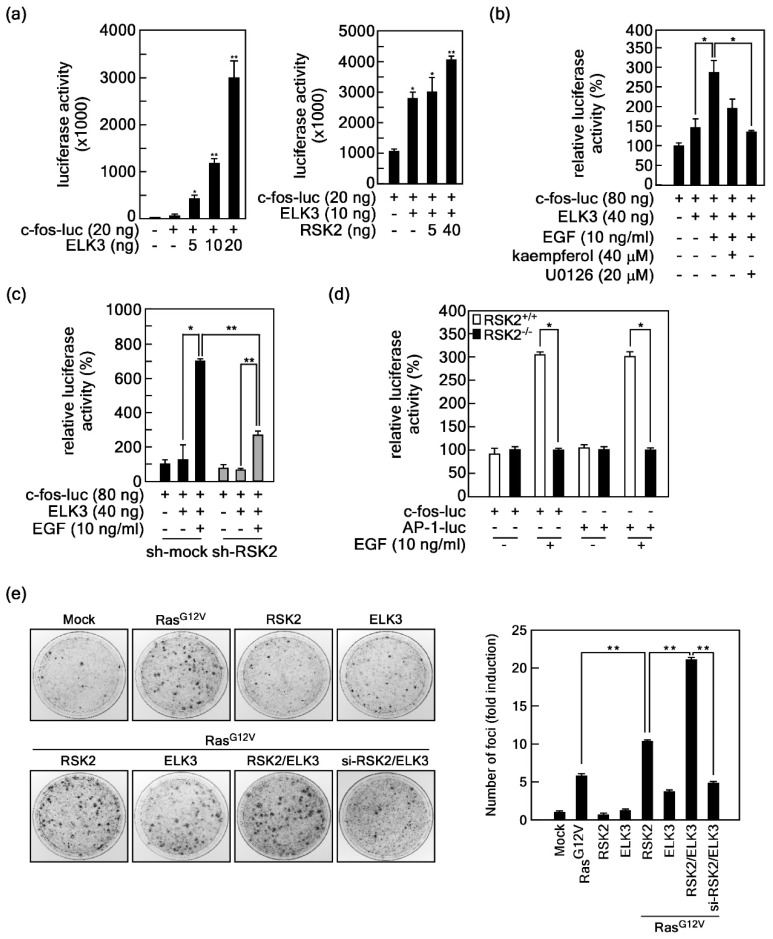
RSK2-mediated ELK3 transactivation activity regulates *c-fos* promoter activity-mediated AP-1 transactivation activity. (**a**) *Left graph*, ELK3 activates *c-fos* promoter activity. The *c-fos*-luciferase reporter plasmids were introduced into HEK293T cells with indicated doses of ELK3 and 80 pg of *phRL*-luciferase expression vector. *Right graph*, ELK3 transactivation activity is regulated by RSK2. The *c-fos*-luciferase reporter plasmids and ELK3 expression vectors were introduced into HEK293T cells with indicated doses of RSK2 expression vector and 80 pg of *phRL-luciferase* expression vector. (**b**) ELK3 transactivation activity was inhibited by inhibition of MEKs-ERKs-RSK2 signaling. The *c-fos*-luciferase reporter plasmid and ELK3 expression vector were introduced into HEK293T cells as indicated. Inhibitory effects of kaempferol, an RSK2 inhibitor, and U0126, a MEK inhibitor, on the transactivation activity of ELK3 induced by EGF were analyzed by examining luciferase activity. (**c**,**d**) Blockage of RSK2 suppresses ELK3-mediated *c-fos* promoter activity and AP-1 transactivation activity. (**c**) RSK2 knockdown suppressed EGF-induced ELK3 transactivation activity. The *c-fos*-promoter luciferase reporter plasmids and ELK3 expression vectors were introduced into sh-mock and sh-RSK2 stable cells and stimulated with EGF as indicated. (**d**) RSK2 knockout suppressed *c-fos* promoter activity and AP-1 transactivation activity. The *c-fos-* and AP-1-luciferase reporter plasmids were introduced into RSK2^+/+^ and RSK2^−/−^ mouse embryonic fibroblasts and stimulated with EGF as indicated. (**e**) Representative images showing the effects of RSK2-mediated ELK3 transactivation activity and Ras^G12V^-mediated cell transformation. Constitutive active Ras (Ras^G12V^), RSK2, ELK3, and si-RSK2 were introduced into NIH3T3 cells for assaying of foci formation as indicated. The cells were cultured for 10–14 days to form foci by abrogation of cell-cell contact inhibition. The formed foci were visualized by crystal violet staining. Formed foci numbers were counted using the NIH ImageJ computer program (Version 1.6). Fold induction is expressed relative to that of NIH3T3-mock. (**a**–**d**) The firefly luciferase activity was normalized to *Renilla* luciferase activity. Data were obtained from three independent experiments, and the values presented are means ± SEM. * *p* < 0.05; ** *p* < 0.01 versus an indicated control group (Student’s *t*-test).

Since phosphorylation is a key posttranslational modification for activity regulation of transcription factors, we also searched for the putative phospho-motif for RSK2 in ELKs. Putative phosphorylation motifs for RSK2, RxRxxS/T or RxxS/T, were found at two sites at the junction of variable domain I and the B domain, two sites at the N-terminal inhibitory domain (NID), two sites at variable domain II, one site at the C domain, and two sites at variable domain III (Supplementary Figures S1 and S2a). Interestingly, the serine residue at 363 of ELK3, which is a corresponding amino acid of ELK1 at Ser389 and an ERK-mediated phosphorylation target, was a component of the conserved RSK2 target phosphorylation motif (Supplementary Figures S1 and S2a,b). Therefore, we analyzed transactivation activity using the *c-fos* promoter luciferase (*c-fos-luc*) reporter assay (Supplementary Figure S3a). We found that *c-fos* promoter-mediated luciferase activity was increased by ELK3 expression in a dose-dependent manner (Figure 3a, left graph). To examine the effects of RSK2 on the ELK3-mediated *c-fos* promoter activity, we co-expressed RSK2 and ELK3 and observed that ELK3-induced *c-fos* promoter activity was enhanced by co-expression with RSK2 in a dose-dependent manner (Figure 3a, right graph). Notably, ELK3-induced *c-fos* promoter activity, which was increased by EGF stimulation, was abrogated by treatment of kaempferol, a flavonoid known as an RSK2 selective inhibitor [38], and by U0126, a MEK inhibitor [43] (Figure 3b). Importantly, RSK2-mediated ELK3 transactivation activity regulation was confirmed, by which RSK2 knockdown (Supplementary Figure S3b) abolished EGF-induced *c-fos* promoter activity in HEK293T cells (Figure 3c). Similarly, EGF-induced *c-fos* promoter and AP-1 (a dimer of c-Fos and c-Jun) transactivation activities in RSK2^+/+^ mouse embryonic fibroblasts (MEFs) were abrogated by RSK2^−/−^ MEFs (Figure 3d). To examine the role of RSK2-ELK3 signaling in cell transformation, we conducted foci formation assays using NIH3T3 cells. We found that ectopic expression of constitutive active Ras (Ras^G12V^) increased foci formation (Figure 3e). The increased foci numbers by Ras^G12V^ alone were further increased by co-expression of both Ras^G12V^ and RSK2 (Figure 3e). Notably, the foci size was bigger with triple co-expression of Ras^G12V^/RSK2/ELK3 than with Ras^G12V^ alone or with Ras^G12V^/RSK2 (Figure 3e). Importantly, blockage of Ras^G12V^/RSK2/ELK3 signaling by si-RSK2 totally abrogated foci formation (Figure 3e). Additionally, the single expression of RSK2 or ELK3 did not affect foci formation in NIH3T3 cells (Figure 3e). Taken together, these results demonstrated that the RSK2-ELK3 signaling axis has an essential role in growth factor-mediated c-Fos expression, resulting in an increase of AP-1 transactivation activity and cell transformation.

### 2.4. RSK2 Regulates ELK3 Nuclear Localization

**Figure 4:**
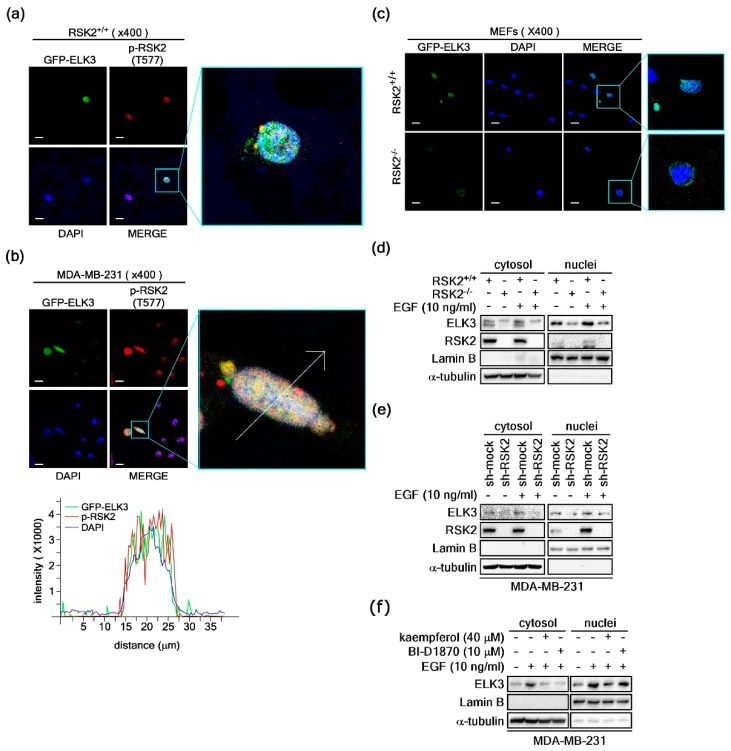
RSK2 mediates nuclear localization of ELK3. (**a**,**b**) Activated RSK2 induces nuclear accumulation of ELK3 in RSK2^+/+^ mouse embryonic fibroblasts (MEFs) (**a**) as well as in MDA-MB-231 cells (**b**). GFP-ELK3 expression vectors were introduced into RSK2^+/+^ MEFs. The active forms of RSK2 were hybridized with RSK2-Thr577 and Alexa 568 conjugated secondary antibodies. Localization of GFP-ELK3 and the active form of RSK2 were visualized under a confocal microscope (×400). DAPI was used for nuclear staining. Scale bars, 20 μm. (**c**) RSK2 deficiency abolished ELK3 nuclear accumulation. GFP-ELK3 expression vectors were introduced into RSK2^+/+^ and RSK2^−/−^ MEFs and cellular distribution of ELK3 was observed under a fluorescence microscope. DAPI was used for nuclear staining. Scale bars, 20 μm. (**d**) ELK3 protein was decreased in nucleus and cytosol in RSK2^−/−^ MEFs. Nuclear and cytosol fractions from RSK2^+/+^ and RSK2^−/−^ MEFs were prepared, and ELK3 protein levels were visualized by western blotting using antibodies as indicated. (**e**) RSK2 knockdown decreased ELK3 protein levels in both nucleus and cytosol. MDA-MB-231 cells stably expressing sh-mock and sh-RSK2 were stimulated with EGF and the endogenous ELK3 protein levels were visualized by western blotting using specific antibodies as indicated. (**f**) Chemical inhibition of RSK2 activity suppresses ELK3 nuclear accumulation. The MDA-MB-231 cells were pretreated with kaempferol and BI-D1870, as indicated, for 30 min prior to EGF stimulation. ELK3 protein levels in the nucleus and cytosol were visualized by western blotting using specific antibodies as indicated.

To examine the role of RSK2/ELK3 signaling in cancer cells, we analyzed the expression profiles of RSK1–RSK4 in cancer tissues within the UniGene database (https://www.ncbi.nlm.nih.gov/unigene). The results showed that cancer types expressing RSK2 at the highest rate compared to the other RSK family members were bladder carcinoma, breast tumor, colorectal tumor, esophageal tumor, liver tumor, prostate tumor, skin tumor, and uterine tumor (Supplementary Figure S4a). In addition, we analyzed the expression profiles of ELKs in cancer tissues in the UniGene database and observed that ELK1 is a major expression type in gastrointestinal tumor, lymphoma, pancreatic tumor, primitive neuroectodermal tumor, retinoblastoma, and soft tissue/muscle tissue tumor (Supplementary Figure S4b). ELK3 was highly expressed in breast tumor, esophageal tumor, head and neck tumor, and liver tumor (Supplementary Figure S4b). ELK4 was expressed as a major type among the ELK profiles in bladder carcinoma, cervical tumor, chondrosarcoma, colorectal tumor head and neck tumor, kidney tumor leukemia, lung tumor, lymphoma, ovarian tumor, prostate cancer, skin cancer, and soft tissue/muscle tissue tumor (Supplementary Figure S4b). Additionally, we used western blotting to analyze the cellular content of ELK3 using HepG2 and MDA-MB-231 breast cancer cells. The results showed that HepG2 cells barely expressed ELK3, whereas MDA-MB-231 cells expressed detectable levels of ELK3 (data not shown). Based on Supplementary Figure S4a and the levels of Figure S4b and ELK3 expressions, we decided to use MDA-MB-231 breast cancer cells in this study. To confirm the subcellular localization of active RSK2 (phospho-RSK2-Thr577) and ELK3, we conducted immunocytofluorescence analysis using RSK2-Thr577 antibody and GFP-ELK3 in RSK2^+/+^ MEFs. The results showed that the active form of RSK2 was mainly observed in the nucleus (Figure 4a). As expected, ELK3 was observed in the nucleus (Figure 4a). Similar to the localization in the RSK2^+/+^ MEFs, MDA-MB-231 cells showed nuclear localization of active form of RSK2 (Figure 4b). Moreover, overlapping of active RSK2 and ELK3 was shown in the confocal microscopy results (Figure 4b). Our previous study demonstrated that RSK2-mediated phosphorylations of p53 and NFAT3 influenced their subcellular localizations [42,44]. Since ELK3 also functions as a transcription factor, we hypothesized that RSK2-mediated ELK3 phosphorylation might affect the subcellular localization of ELK3. To examine this hypothesis, we ectopically overexpressed GFP-ELK3 in RSK2^+/+^ and RSK2^−/−^ MEFs (Figure 4c). The results showed that ELK3 was mainly localized in the nucleus of RSK2^+/+^ MEFs. Importantly, the GFP-ELK3 observed in the nucleus of RSK2^+/+^ MEFs were redistributed mainly to the cytoplasm in RSK2^−/−^ MEFs, indicating that RSK2-mediated activation signaling induces nuclear localization of ELK3 (Figure 4c). The phenomenon was verified by performing western blotting using nuclear and cytoplasm fractions, indicating that an RSK2 deficiency reduces nuclear ELK3 protein levels (Figure 4d). These inhibitory effects of RSK2 on the nuclear localization of ELK3 were also confirmed by the results obtained following RSK2 knockdown (Figure 4e), as well as by chemical inhibition of RSK2 using kaempferol and BI-D1870 (Figure 4f), in MDA-MB-231 breast cancer cells. These results demonstrated that the activation signal of RSK2 regulates nuclear localization of ELK3.

### 2.5. RSK2/ELK3 Signaling Axis Regulates MDA-MB-231 Breast Cancer Cell Growth

**Figure 5:**
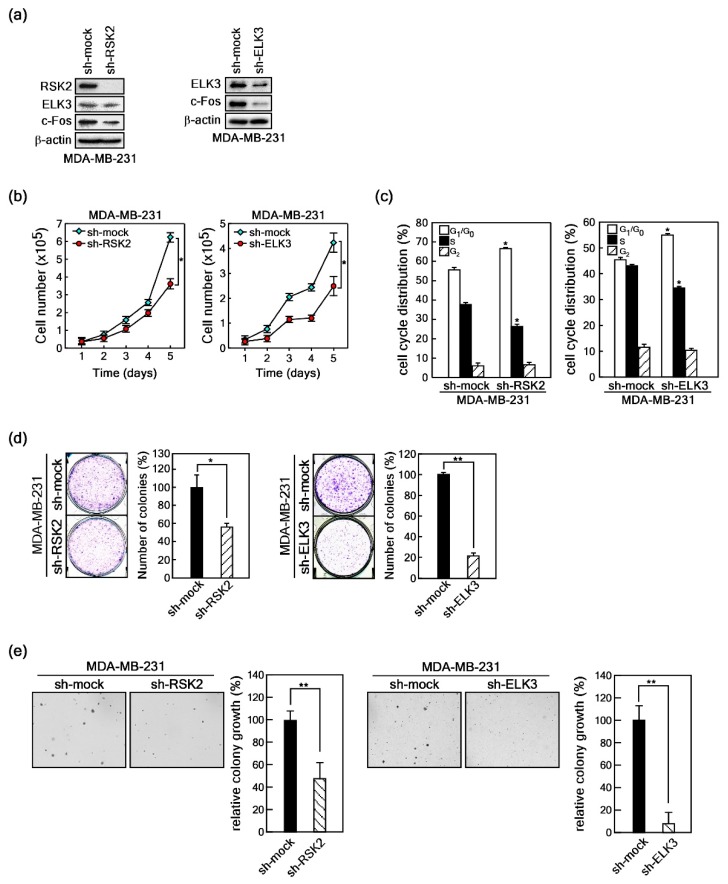
RSK2 and ELK3 are required for MDA-MB-231 cell growth. (**a**) The sh-mock, sh-RSK2 or sh-ELK3 viral particles were separately infected into MDA-MB-231 cells and the puromycin resistance cells were pooled. RSK2 and ELK3 knockdowns were confirmed by western blotting using specific antibodies as indicated. (**b**) Knockdowns of RSK2 or ELK3 inhibit cell proliferation of MDA-MB-231 cells. MDA-MB-231 cells stably expressing sh-RSK2 or sh-ELK3 underwent cell proliferation for 5 days, and cell proliferation was monitored by counting cells using an automated cell counter. (**c**) Knockdowns of RSK2 or ELK3 induce G1/G0 cell-cycle accumulation in MDA-MB-231 cells. MDA-MB-231 cells stably expressing sh-RSK2 or sh-ELK3 were subjected to cell cycle analysis using flow cytometry. (**d**) Knockdowns of RSK2 or ELK3 inhibit foci formation in MDA-MB-231 cells. MDA-MB-231 cells stably expressing sh-RSK2 or sh-ELK3 underwent foci formation for 9-10 days. The formed foci were visualized by crystal violet staining and counted using the NIH ImageJ computer program (Version 1.6). (**e**) Knockdowns of RSK2 or ELK3 inhibit anchorage-independent colony growth of MDA-MB-231 cells. MDA-MB-231 cells stably expressing sh-RSK2 or sh-ELK3 underwent anchorage-independent colony growth assay (soft agar assay). The colony numbers were observed under an ECLIPSE Ti inverted microscope and counted using the NIS-Elements AR (V. 4.0) computer software program. (**b**–**e**) Data were obtained from three independent experiments, and the values presented are means ± SEM. * *p* < 0.05, ** *p* < 0.01 versus the indicated control group (Student’s *t*-test).

To examine the phenotypes for RSK2-mediated ELK3 transactivation regulation, we first established stable expressions of sh-RSK2 and sh-ELK3 in MDA-MB-231 cells (Figure 5a). Using these cells, we observed that knockdown of RSK2 or ELK3 suppressed cell proliferation of MDA-MB-231 cells (Figure 5b). Based on cell cycle analysis, we confirmed that RSK2 or ELK3 knockdown increased the size of the cell population in the G1 phase of the cell cycle and decreased the population in the S phase of the cell cycle of MDA-MB-231 cells (Figure 5c). Next, we explored the capacity for cancer cell colony growth by undertaking foci formation assays in MDA-MB-231 cells. Similar to the results of the cell proliferation analysis, knockdown of RSK2 or ELK3 suppressed foci formation in MDA-MB-231 cells (Figure 5d). Since cancer cells generally grow in anchorage-independent conditions, we examined the knockdown effects of RSK2 and ELK3 on colony growth in soft agar. We found that knockdown of RSK2 or ELK3 significantly inhibited MDA-MB-231 cell colony growth in soft agar (Figure 5e). Taken together, the results demonstrated that the RSK2/ELK3 signaling axis has an essential role in MDA-MB-231 cell proliferation and colony growth.

## 3. Discussion

Abnormal behavior of healthy cells, such as excessive and uncontrolled cell proliferation, is a well-known characteristic of cancer cells. Abnormal intra- and extra-signaling networks are driven by the dysregulation of one or more signaling networks caused by genetic aberrations including mutations and chromosomal rearrangements [45]. Thus, cancers can develop in a cellular context, which is determined by the dynamics of the underlying signaling networks and the environment [46,47]. Activation of receptor tyrosine kinases, such as by epidermal growth factor receptor, by receptor-ligand interaction outside of the cytoplasmic membrane is an initiation point in the production of diverse cellular activation signaling pathways that regulate ultimate cellular processes including cell proliferation, survival, differentiation, apoptosis, and cancer development [48,49,50]. For example, signaling molecules of the RAS-ERK signaling pathway are highly mutated in human cancer. Thus, activation of Ras-Rafs-MEKs-ERKs in human cancers results in the activation of downstream kinases of ERKs such as p90 ribosomal S6 kinases (p90RSKs; RSKs) including RSK2, indicating that RSKs are key effector kinases enhancing cell proliferation and transformation [49,50]. Since the RSK family has very high amino acid homology, especially in the N- and C-terminal kinase domains, and as the family is well conserved in different species, we believe that RSKs are functionally well-conserved kinases that have a key role in the transduction of activation signals to the nucleus in the Ras-mediated signaling pathway.

Over the last decade, our research group has investigated the physiological roles of RSKs including RSK1, 2, 3, and 4 and mitogen- and stress-activated protein kinase 1-2 (MSK1-2) as downstream signaling molecules of ERKs. It has been shown that RSK2 deficiency, but not other RSK and MSK members, causes Coffin-Lowry Syndrome, an X-linked genetic disorder marked by cognitive disabilities, short stature, skeletal abnormalities, and abnormal characteristics of the face, trunk, and limbs in humans [51]. These results clearly indicated that the physiological roles of RSK2 are not redundant among those of other isotypes of RSKs under physiological conditions [49]. However, questions about how mutation(s) in one gene exhibit(s) diverse phenotypes in humans have not yet been fully elucidated. One possible suggestion made by our research group is that RSK2 might have multiple binding partners [49]. Such multiple binding partners can act as RSK2 substrates, and their activities can be regulated according to the physiological context [49]. We recently summarized the role of RSK2 and its binding partners in cancer development [49]. Based on these background findings, we sought to identify novel RSK2 binding partners. We determined that ELKs, which are downstream transcription factors of ERKs and p38 kinases, are downstream of RSKs including RSK2 (Figure 1 and Figure 2). That finding was critically important because RSK2 is emerging as a signaling molecule that has key roles in cell proliferation and transformation [38], cell migration and invasion [37], cancer cell growth [25], and stress tolerance and chemoresistance [39]. Taken together, studies on the role of the RSK2-ELK3 signaling axis in carcinogenesis might provide novel opportunities for elucidating the molecular mechanisms involved in cancer development.

For the last two decades, our research group has studied the protein–protein interactions of RSK2. Activation of RSK2 is triggered by the binding of growth factors to specific receptors at the cytoplasmic membrane, resulting in dimerization of specific receptors and provocation of activation signals by phosphorylation at tyrosine residues [49]. Once activated, the activation events transduce to transcription factors via downstream signaling molecules such as Raf, MEKs, ERKs, and RSKs. For the last 17 years, our research group has investigated the signaling network and physiological function of RSK2. Since we hypothesized that the roles of RSK2 and RSK2-mediated cellular phenotypes are determined by protein–protein interactions, we extensively screened numerous transcription factors to identify the binding partners. Since RSK2 deficiency attenuates cell proliferation and EGF- or TPA-induced anchorage-independent cell transformation [38], an initial conclusion regarding RSK2 physiological function is that it can induce cell survival. Based on the accumulation of interaction-dependent RSK2 signaling axes, we have determined that RSK2 is a multifunctional signaling molecule involved in various cellular processes including: cell survival by modulation of cell survival/apoptosis-related proteins such as BAD, Caspase-8, and GSK3β; chromatin remodeling by phosphorylation of epigenetic factors such as p53, histone H3, H2B, and H2AX; cell cycle regulation and cell transformation by induction of transactivation activity factors such as ATF1, CREB2, and c-Fos; and, immune modulation by regulation of transactivation activity factors such as NFAT3, and NF-κB [49]. In this study, we uncovered a novel signaling axis, RSK2-ELK3, and provided several important results from MDA-MB-231 cell assays showing that the RSK2-ELK3 signaling axis has an important role in breast cancer cell proliferation and colony growth.

## 4. Materials and Methods

### 4.1. Chemicals and Antibodies

Chemicals such as Tris, NaCl, and SDS for molecular biology and buffer preparation were purchased from Sigma-Aldrich (St. Louis, MO, USA). Media for cell culture, such as Dulbecco’s modified Eagle’s medium (DMEM; Cat#: 10-013-CVR, Corning, New York, NY, USA), and other supplements were obtained from Life Science Technologies (Rockville, MD, USA) or Corning. Antibodies for analysis of western blotting, immunoprecipitation, and immunocytofluorescence assays, including Gal4 tag (Cat#: SC-510 and SC-510 HRP), VP-16 tag (Cat#: SC-7546 and SC-7546 HRP), c-Myc tag (Cat#: SC-40 and SC-40 HRP), GST tag (Cat#: SC-138), RSK2 (Cat#: SC-9986), Lamin B (Cat#: SC-6216), α-tubulin (Cat#: SC-8035), phospho-RSK2-Thr577 (Cat#: SC-16407) and β-actin (Cat#: SC-69879), were purchased from Santa Cruz Biotechnology (Santa Cruz, CA, USA). Antibodies against His tag (Cat#: R941-25) and ELK3 (Cat#: PA5-35173) were purchased from Thermo Fisher Scientific (Waltham, MA, USA). Antibodies for phospho-RxxS/T (Cat#: 9614) and c-Fos (Cat#: 4384) were purchased from Cell Signaling Biotechnology (Beverly, MA, USA). Antibody against phospho-Ser/Thr (ab17464) was purchased from Abcam (Cambridge, UK). Human recombinant EGF was purchased from BD Sciences (San Jose, CA, USA). Dimethyl sulfoxide (DMSO, Cat#: D8418, Sigma-Aldrich) and kaempferol (Cat#: ALX-385-005, Enzo, Farmingdale, NY, USA) were purchased from Sigma-Aldrich. The U0126 (Cat#: 9903) was obtained from Cell Signaling Biotechnology (Beverly, MA, USA), and BI-D1870 (Cat#: S2843) was obtained from Selleckchem (Houston, TX, USA). Active RSK2 recombinant protein (Cat#: 7768) was purchased from BioVision (Milpitas, CA, USA). RNase A (Cat#: R5125) and propidium iodide (Cat#: P4170) were purchased from Sigma-Aldrich.

### 4.2. Cell Culture and Transfection

RSK2^+/+^ and RSK2^−/−^ MEFs were a generous gift from Dr. J.C. Brüning (Institute for Genetics, Center for Molecular Medicine Cologne, Cologne, Germany) and were cultured in DMEM supplemented with 10% fetal bovine serum (FBS) and antibiotics. The HEK293T, NIH3T3, and MDA-MB-231 cell lines were purchased from the American Type Culture Collection (Manassas, VA, USA). HEK293T and MDA-MB-231 cells were cultured in DMEM supplemented with 10% FBS and antibiotics. NIH3T3 cells were cultured in 10% calf serum-DMEM and antibiotics. All cells were maintained at 37 °C in a 5% CO_2_ incubator and were split at 90% confluence. The media were exchanged every 2 or 3 days. When the cells reached 60% confluence, they were transfected with expression vectors by using jetPEI (Polyplus-Transfection, New York, NY, USA) according to the manufacturer’s instructions.

### 4.3. Mammalian Two-Hybrid Assay

To screen for protein-binding partners, we conducted mammalian two-hybrid assays in accordance with the Promega Checkmate mammalian two-hybrid system protocols (Promega, Madison, WI, USA). HEK293T cells (2 × 10^4^ cells/well) were seeded into 48-well plates and maintained with 10% FBS-DMEM for 18 h before conducting transfection. The vectors, pACT-transcription factors consisting of ELKs (ELK1, ELK3, and ELK4), pBIND-RSK2, and pG5-luciferase were mixed at the same molar ratios (1:1:1), and the total amount of DNA was not more than 100 ng per well. Transfection was done utilizing jetPEI according to the manufacturer’s recommendations. For the luciferase assay, the cells were disrupted by directly adding cell lysis buffer and gently shaking for 30 min at room temperature (RT) and then 60 μL aliquots were added to each well of a 96-well luminescence plate. Luminescence activity was automatically measured through a VICTOR X3 plate reader (PerkinElmer, Waltham, MA, USA). To evaluate transfection efficiency, relative luciferase activity was calculated based on the pG5-luciferase basal control and was normalized against *Renilla* luciferase activity, which was included in the pBIND vector.

### 4.4. Western Blotting

To obtain proteins for western blotting, cells were lysed by freezing and thawing with NP-40 cell lysis buffer (150 mM NaCl, 40 mM Tris pH 8.0, and 0.5% NP-40; Roche protease inhibitor cocktail); after which, the supernatant was recovered by centrifugation at 4 °C. Using the DC protein assay kit (Bio-Rad Laboratories, Hercules, CA, USA), the protein concentration was determined. Equal amounts of protein were resolved by performing 10–15% SDS-PAGE and transferred to polyvinylidene difluoride (PVDF, Merck Millipore Ltd., Burlington, MA, USA) membranes. The membranes were blocked with 5% skim milk/1× PBS containing 0.05% Tween 20 (PBS-T) and hybridized with specific antibodies as indicated. Western blots were visualized by using an enhanced chemiluminescence detection system (Amersham Biosciences, Piscataway, NJ, USA) and a Chemidoc XRS imager system (Bio-Rad Laboratories).

### 4.5. Immunoprecipitation

HEK293T and MDA-MB-231 cells (2 × 10^6^) were seeded into 100 mm dishes and incubated overnight. Individual expression vectors were transfected into HEK293T or MDA-MB-231 cells with jetPEI, and the cells incubated for 24 h. Protein samples from incubated cells were extracted using NP-40 cell lysis buffer; subsequent immunoprecipitation was conducted with the same amount of each sample and the antibody specific to the vector-transfected sample. The protein extracts were combined with protein G beads (50% slurry) (Cat#: 17-0618-02, Protein G Sepharose 4 Fast Flow, GE Healthcare, Little Chalfont, UK) by rocking at 4 °C for at least 5 h or overnight. The protein G beads were washed and mixed with 6× SDS sample buffer and boiled. The precipitated proteins were resolved by 10–15% SDS-PAGE and detected by immunoblotting with specific antibodies as indicated.

### 4.6. Gene Silencing

To construct RSK2 or ELK3 knockdown in HEK293T and MDA-MB-231 cells, lentiviral expression plasmids of pLenti-sh-RSK2 and -sh-ELK3 (Dharmacon, Lafayette, CO, USA) were co-transfected into HEK293T cells with psPAX2 and pMD2.G (Addgene, Cambridge, MA, USA) as indicated by the manufacturer’s recommended protocols. At 24 h and 48 h after transfection, we obtained a Lenti-sh-RSK2 and Lenti-sh-ELK3 medium containing viral particles from the HEK293T cells. The medium was filtered with a 0.45 μm filter (Cat#: 723-2545, Thermo Fisher Scientific) and used, with 1–2 μg/mL of polybrene, to infect HEK293T and MDA-MB-231 cells. After a maximum of 16 h, the cell medium was exchanged with fresh complete medium. After 48 h of maintenance, non-infected control cells were killed over a period of 3 days by treatment with 2 μg/mL of puromycin (Cat#A111308, Thermo Fisher Scientific). Surviving cells were immediately examined to determine cell proliferation, cell cycle phases, cell transformation, and luciferase activity.

### 4.7. Cell Cycle Analysis

MDA-MB-231 cells (4 × 10^5^) were seeded into 60 mm dishes and maintained for 48 h at 37 °C in a 5% CO_2_ incubator. For cell cycle analysis, cells were trypsinized, washed with ice-cold 1× PBS, and fixed with ice-cold 70% ethanol over a period of at least 2 h. The cells were treated with RNase A (200 μg/mL; Sigma-Aldrich) for 40 min at RT and propidium iodide (PI; 20 μg/mL; Sigma-Aldrich) for 15 min at 4 °C. The cells were analyzed for cell cycle phase by performing flow cytometry on a BD FACSCalibur™ flow cytometer (BD, Franklin Lakes, NJ, USA).

### 4.8. Reporter Gene Assay

HEK293T cells (2 × 10^4^ cells/well) were seeded into 48-well cell culture plates and maintained with 10% FBS-DMEM for 18 h before transfection. The cells were transiently co-transfected with 20 ng of a *c-fos* promoter luciferase plasmid and pACT-ELK3 with/without pcDNA4-HisMax-RSK2 at the indicated doses and with 80 pg of the *phRL-SV40 Renilla* luciferase reporter plasmid. For treatment of inhibitors, including kaempferol and U0126, induced by EGF, the cells were cultured for 24 h after transfection and then starved in serum-free medium overnight. The cells were pretreated with the indicated doses of kaempferol or U0126 and then co-treated with EGF (10 ng/mL) at the indicated inhibitor doses for 3 h. The HEK293T stably expressing sh-mock and sh-RSK2 as well as RSK2^+/+^ and RSK2^−/−^ MEFs underwent the same procedures. Finally, the cells were disrupted, and the level of firefly luciferase activity was measured through a VICTOR X3 plate reader (PerkinElmer). Firefly luciferase activity was normalized by *Renilla* luciferase activity to determine transfection efficiency.

### 4.9. In Vitro Kinase Assay

A GST-ELK3 protein (500 ng) purified from *Escherichia coli* was used as a substrate for in vitro kinase assay and was combined with active RSK2 (50 ng or 100 ng). The assay reaction was processed at 30 °C for 30 min in a reaction mixture comprised of 100 μM of cold ATP. The reaction was stopped by adding 6× SDS sample buffer followed by boiling. The proteins were resolved by 8%–10% SDS-PAGE. The ELK3 phosphorylation by active RSK2 was visualized by western blotting using phospho-Ser/Thr specific antibody as indicated.

### 4.10. Vector Construction

Serial deletion mutants of RSK2 were constructed as described previously [42]. To construct VP16-ELK3 serial deletion mutants, the cDNA fragment was amplified by PCR with sense primers (a) ELK3-D1 5′-GCGTCGACAAATGGAGAGTGCAATCACGCTG-3′ (b) ELK3-D2 : 5′- GCGTCGACAAATGGATCCTCACGCGGTGGAG-3′ (c) ELK3-D3 : 5′- GCGTCGACAATCCGCCTTCCTGGCCTCGTCC-3′ (d) ELK3-D4 : 5′- GCGTCGACAACCCCCAAAGGCCAAAAAACCC-3′ (with the Sal I site underlined) and antisense primers (a) ELK3-D1 : 5′-TTGCGGCCGCTCACTTCAGGATCTCCGGGAA-3′ (b) ELK3-D2 : 5′-TTGCGGCCGCTCACGCCGCAGCCTCTGA-3′ (c) ELK3-D3 : 5′-TTGCGGCCGCTCAAAGAGATGGAGACTTGGT-3′ (d) ELK3-D4 : 5′-TTGCGGCCGCTCAGGATTTCTGAGAGTTTGA-3′ (with the Not I site underlined). The cDNA fragment was introduced into the Sal I/Not I sites of pACT and constructed as pACT-VP16-ELK3-FL, -D1, -D2, -D3, and -D4. All vectors were confirmed by restriction mapping and DNA sequencing.

### 4.11. Immunocytofluorescence Assay

RSK2^+/+^ cells (1 × 10^4^ cells/well) and MDA-MB-231 cells (3 × 10^4^ cells/well) were seeded into four-chamber slides and transfected with pEGFP-ELK3. After 48 h of incubation, the cells were washed and fixed with 4% formalin. Then, the cells were permeabilized using 0.5% Triton X-100/1× PBS, blocked with 1× PBS/0.02% Tween20/1% BSA at 37 °C for 1 h, and hybridized with the indicated antibodies against phospho-RSK2-Thr 577 overnight at 4 °C, then hybridized with anti-goat-Alexa-568 (Cat#: A-11057, Thermo Fisher Scientific) at RT for 1 h. The GFP fusion proteins and the RSK2 protein levels were visualized using a LSM 710 laser scanning confocal microscope (Carl Zeiss Korea Co. Ltd., Seoul, Korea).

### 4.12. Cell Count Assay

MDA-MB-231 stably expressing sh-mock and sh-RSK2 or sh-ELK3 cells (3 × 10^4^ cells/well) were seeded into a 6-well plate and incubated for the indicated times. Then, the medium was removed, and the cells were trypsinized. After staining with trypan blue, the number of cells was counted in a cell-counting slide of a cell counter (Bio-Rad TC20™ automated cell counter, Hercules, CA, USA) over a period of 5 days at 24 h intervals.

### 4.13. Anchorage-Independent Colony Growth Assay

The soft agar assay was performed with MDA-MB-231 cells stably expressing sh-RSK2 or sh-ELK3 cells. The cells (8 × 10^3^/well) were suspended in 10% FBS-DMEM, added to 0.3% agar, and incubated at 37 °C in a 5% CO_2_ incubator for 20–25 days. At that time, the cell colonies were counted using an ECLIPSE Ti inverted microscope (Nikon Instruments Korea, Gangnam, Seoul, Korea) and NIS-Elements AR (V. 4.0) computer software as described previously [52].

### 4.14. Ni-IDA Pulldown Assay

Each of the His-ELK3-FL, -D1, -D2, -D3 or -D4 fusion proteins coupled with His-Bind Agarose Resin (Ni-IDA) (ELPIS BIOTECH, Taejeon, Korea) were incubated at 4 °C for 30 min with suspension buffer (150 mM NaCl, 40 mM Tris pH 8.0 and 0.5% NP-40) and cell lysate (400 μg) of HEK293T cells expressing Myc-RSK2. The beads were washed three times with wash buffer (100 mM NaCl, 20 mM Tris pH 8.0, 1 mM EDTA and 0.5% NP-40) at 4 °C, then mixed with 6× SDS sample buffer and boiled. The interacted proteins were visualized by western blotting using Myc tag and RSK2 antibodies as indicated.

### 4.15. Statistical Analysis

All data were obtained from three independent experiments. Data are presented as mean ± SEM values. The Student’s *t*-test was used to compare values between two groups. *p*-values < 0.05 (two-tailed) were considered significant.

## 5. Conclusions

The finding of a novel signaling axis in this study is very important to furthering the understanding of the carcinogenesis process during cancer development. We determined that ELKs, including ELK3, are direct downstream target transcription factors of RSK2 that are activated by interactions between the linker domain of RSK2 and the D and C domains of ELK3. The interaction of RSK2 and ELK3 regulates *c-fos* promoter activity, resulting in modulation of AP-1 transactivation activity. The RSK2-ELK3 signaling axis was not only observed in premalignant cells, but also in MDA-MB-231 breast cancer cells. These results of this study indicate that the novel RSK2/ELK3 signaling axis, by enhancing c-Fos-mediated AP-1 transactivation activity, has an essential role in cancer cell proliferation and colony growth.

## Supplementary Materials

Supplementary Materials can be found at https://www.mdpi.com/1422-0067/20/8/1994/s1.
