# Supplementary material for: RSK2-Mediated ELK3 Activation Enhances Cell Transformation and Breast Cancer Cell Growth by Regulation of c-fos Promoter Activity
Source: Int J Mol Sci. 2019 Apr 23;20(8):1994. doi: 10.3390/ijms20081994 (PMC6515335; doi:10.3390/ijms20081994)
Supplement: Supplementary file 1 [file ijms-20-01994-s001.pdf]

[Supplementary Information]

RSK2-mediated ELK3 activation enhances cell transformation and breast cancer cell growth by regulation of c-fos promoter activity

Sun-Mi Yoo[1], Cheol-Jung Lee[1], Hyun-Jung An[1], Joo-Young Lee[1], Hye-Suk Lee[1], Han Chang Kang[1], Sung-Jun Cho[2], Seung-Min Kim[1], Juhee Park[1], Dae-Joon Kim[3], Yong-Yeon Cho[1,*]

Contents:

1. Supplementary Table legends: S1
2. Supplementary Figure legends: S1-S4

Supplementary Table 1

normal tissues							
tissues	RSK1	RSK2	RSK3	RSK4	ELK1	ELK3	ELK4
adipose tissue	0	0	0	0	0	0	0
adrenal gland	30	30	91	0	0	60	30
ascites	125	125	50	0	25	0	25
bladder	0	33	0	0	33	0	66
blood	163	32	24	0	8	0	8
bone	27	83	27	0	13	0	69
bone marrow	143	61	0	0	0	41	41
brain	17	9	110	6	16	20	31
cervix	103	20	0	0	61	20	41
connective tissue	53	26	73	6	6	26	26
ear	0	62	0	0	0	62	0
embryonic tissue	42	51	32	4	18	23	37
esophagus	49	49	0	0	0	99	0
eye	38	28	95	23	52	4	62
heart	33	22	44	0	0	55	55
intestine	94	34	8	0	12	17	30
kidney	14	56	33	23	0	0	99
larynx	0	0	85	0	0	0	0
liver	38	82	4	0	4	48	29
lung	77	47	50	17	20	29	59
lymph	361	0	0	0	22	0	45
lymph node	122	22	22	0	11	144	100
mammary gland	26	59	105	6	6	39	52
mouth	105	45	30	0	0	90	45
muscle	37	112	65	0	0	18	75
nerve	0	0	0	0	0	0	0
ovary	108	39	49	9	29	0	29
pancreas	14	46	42	23	9	4	32
parathyroid	48	48	97	97	0	0	0
pharynx	24	73	0	0	0	49	24
pituitary gland	0	0	60	0	0	0	0
placenta	14	35	10	3	10	24	17
prostate	42	68	79	5	15	5	89
salivary gland	0	0	98	0	0	0	49
skin	52	75	33	4	9	42	37
spleen	430	74	37	0	0	37	37
stomach	94	62	10	0	31	10	31
testis	41	25	39	16	18	16	29
thymus	188	37	25	0	25	37	75
thyroid	171	128	257	42	42	42	64
tonsil	176	0	0	0	0	0	0
trachea	77	77	96	19	0	154	57
umbilical cord	72	0	72	0	0	145	0
uterus	43	43	25	17	38	103	56
vascular	19	116	38	0	0	232	19

Supplementary Table S1. EST gene expression profiles of RSKs and ELKs. Transcripts amount of human RSKs and ELKs, including RSK1 (Hs.149957), RSK2 (Hs.445387), RSK3 (Hs.655277), RSK4 (Hs.234478), ELK1 (Hs.181128), ELK3 (Hs.46523) and ELK4 (Hs.497520) were downloaded from UniGene (https://www.ncbi.nlm.nih.gov/unigene/?term=) and the approximate gene expression patterns were summarized. The numbers indicate specific gene transcripts per million counted transcripts. The red color indicates the highest number of EST count for RSKs or ELKs.

Supplementary Figure S1 by Yoo et al

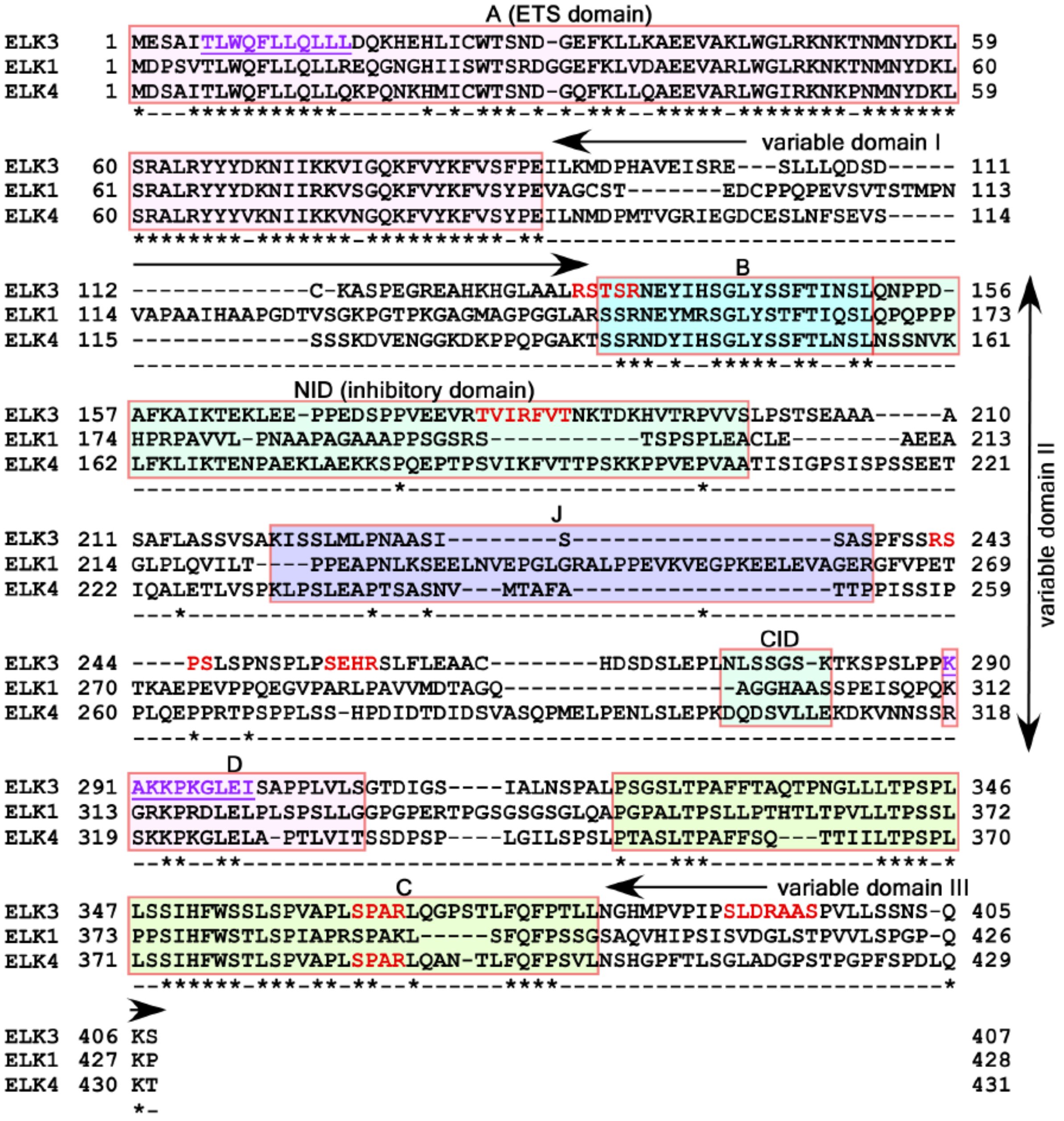

Supplementary Figure S1. Alignment of amino acid sequences of ELK1, ELK3, and ELK4. The A, B, NID, J, CID, D, and C domains of the ELKs are boxed with different colors. Conserved amino acids are noted with star marks in the bottom line. Putative RSK2 phosphorylation motifs in the ELK domains are highlighted as red letters.

Supplementary Figure S2a+S2b by Yoo et al

(a)

S133	127	AALRSTSRNE	136
S131	137	YENRSTSRLA	128
T187	181	TVIRFVTNKT	190
T181	189	TVFRIVTRVE	178
S245	239	FSSRSPSLSP	248
S254	260	FLSRHESPLP	251
S363	369	GQLRAPSLPA	360
S396	390	SLDRAASPVL	399
S390	396	SAARDLSPIP	387

RSK2 phospho-target : RxxS/T
consensus sequence

(b)

GPS analysis of ELK3

Sequence	Position	Score	Cutoff
HGLAALRSTSRNEYI	131	11.147	8.112
LAALRSTSRNEYIHS	133	10.882	8.112
YSSFTINSLQNPPDA	150	9.147	8.112
LMLPNAASISSASPF	232	12.147	8.112
PFSSRSPSLSPNSPL	245	10.382	8.112
SPNSPKPSEHRSLFL	254	8.765	8.112
LEPLNLSSGSKTKSP	278	9.529	8.112
LSPVAPLSPARLQGP	363	13.382	8.112
PSLDRAASPVLLSSN	396	10.618	8.112

RSK2 phospho-target consensus sequence: RxxS/T

Supplementary Figure S2. Summary of putative RSK2 phospho-motifs in ELK3. (a) Amino acid alignment of putative RSK2 phospho-motif, RxxS/T, in ELK3. (b) Group-based prediction system (GPS) analysis of phosphorylation sites. Kinase-specific phosphorylation site prediction analysis was performed by using the GPS against RSK2. (a and b) The analysis results were compared to the RSK2 phospho-motif. Amino acids 131, 133, 245, 254, 363 and 396 matched with (a) and (b) analysis.

Supplementary Figure S3a+S3b by Yoo et al

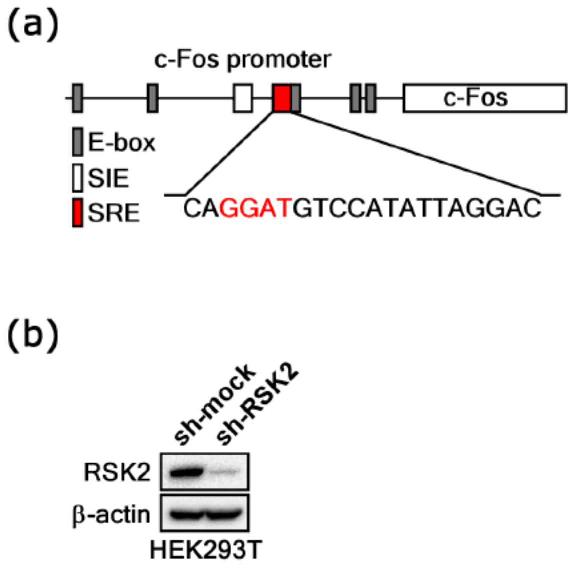

Supplementary Figure S3. *Cis* elements of the *c-fos*-luciferase reporter plasmid and confirmation of RSK2 knockdown. (a) The ELK3 binding consensus nucleotide sequence is highlighted in red. (b) HEK293T cells stably expressing sh-mock or sh-RSK2 were established and RSK2 knockdown was confirmed by the western blotting results. β-actin was used as the internal control for equalized protein loading.

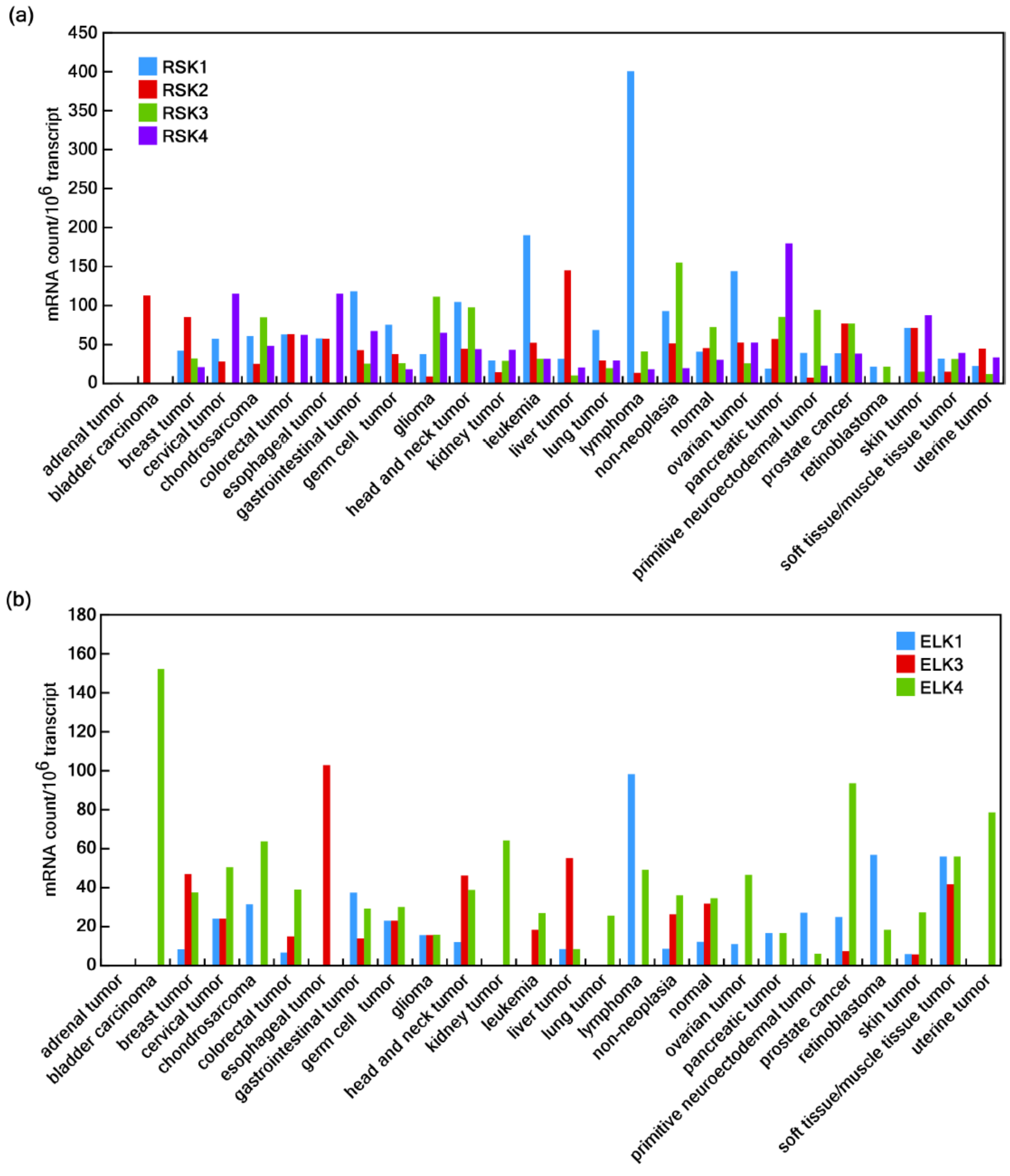

Supplementary Figure S4. EST expression profiles of RSKs and ELK3 in various human cancers. (a) EST expression profiles of RSK1, 2, 3, and 4 in various human cancers were obtained from UniGene (https://www.ncbi.nlm.nih.gov/unigene/?term=). (b) EST expression profiles of ELK1, 3, and 4 in various human cancers were obtained from UniGene (https://www.ncbi.nlm.nih.gov/unigene/?term=).
